# A Review of the Effects of Puerarin on Glucose and Lipid Metabolism in Metabolic Syndrome: Mechanisms and Opportunities

**DOI:** 10.3390/foods11233941

**Published:** 2022-12-06

**Authors:** Xiaoxuan Jing, Jingxuan Zhou, Nanhai Zhang, Liang Zhao, Shiran Wang, Liebing Zhang, Feng Zhou

**Affiliations:** 1 Beijing Key Laboratory of Functional Food from Plant Resources, College of Food Science and Nutritional Engineering, China Agricultural University, Beijing 100083, China; 2 Beijing Engineering and Technology Research Center of Food Additives, Beijing Technology and Business University (BTBU), Beijing 100048, China

**Keywords:** puerarin, sugar metabolism, lipid metabolism, metabolic syndrome, bioavailability

## Abstract

Chronic diseases, including metabolic syndrome related to sugar and lipid metabolic disorders, are the leading causes of premature death around the world. Novel treatment strategies without undesirable effects are urgently needed. As a natural functional ingredient, puerarin is a promising alternative for the treatment of sugar and lipid metabolic disorders. However, the applications of puerarin are limited due to its poor solubility and short half-life. Various drug delivery systems have been investigated to improve the bioavailability of puerarin. This review summarizes the mechanisms involved in the beneficial action of puerarin: suppressing the release of glucose and FFA; regulating the transport of glucose and fatty acids; acting on the PI3K–Akt and AMPK signaling pathways to decrease the synthesis of glucose and fatty acids; acting on the PPAR signaling pathway to promote β-oxidation; and improving insulin secretion and sensitivity. In addition, the preparation technologies used to improve the bioavailability of puerarin are also summarized in this review, in the hope of helping to promote the application of puerarin.

## 1. Introduction

In recent decades, rapid socioeconomic and technological development has led to changes in food supply and dietary patterns [1]. For example, the consumption of sugared beverages and fast food with high fat and high sugar contents has increased markedly [2,3]. According to the data on food availability provided by the Food and Agriculture Organization of the United Nations (FAO), in past the 50 years, the supply quantity of sugar and sweeteners per capita per year has increased by 26.96%, and that of oils and fats has increased by 44.49%. Unhealthy dietary patterns are related to the increasing risk of the metabolic syndrome (MetS), a collection of metabolic disorders typically characterized by type 2 diabetes mellitus (T2DM), hyperlipidemia, fatty liver, and insulin resistance (IR) [4]. The World Health Organization Technical Report indicated that, because of changes in diet, the chronic diseases related to MetS mentioned above are becoming significant causes of premature death around the world [5].

For the treatment of the diseases induced by sugar and lipid metabolic disorders, various kinds of drug have been investigated and used. Metformin is an oral glucose-lowering agent, thiazolidinediones (TZDs) are important insulin sensitizers, and both of these are used in the treatment of T2DM [6,7]. As the rate-limiting enzyme in the cholesterol biosynthesis pathway, 3-hydroxy-3-methylglutaryl-CoA reductase (HMGCR) can be the target of statins. There are literature reports that statins can reduce the level of cholesterol in the body by inhibiting HMGCR [8]. Fibrates are a class of drugs that can effectively reduce lipid accumulation via activation of the hepatic peroxisome proliferator-activated receptors (PPARs), which regulate de novo synthesis of lipids and β-oxidation of fatty acids (FAs) [9]. However, the adverse effects of these drugs remain an ongoing problem. Evidence suggests that up to 25% of patients suffer gastrointestinal side effects associated with metformin [6]. The clinically used TZDs also have some serious side effects, such as hepatotoxicity and weight gain [7]. Statin therapy can cause more serious problems, including new-onset T2DM, hepatotoxicity, and renal toxicity [8]. Fibrates are associated with serum aminotransferase elevation during therapy, which indicates liver injury [9]. Thus, looking for a natural active ingredient with low toxicity and low side effects to improve disorders of glucose and lipid metabolism has become the focus of scholars in this field.

*Pueraria lobata* is a medicinal and edible plant widely distributed in eastern and southern Asia and is one of the earliest herbs used in ancient China [10]. Puerarin is the major isoflavone isolated from the root of *Pueraria lobata* and was first isolated in the late 1950s [11]. Earlier studies have noted that puerarin has great potential effectiveness in the regulation of glucose and lipid metabolic disorders, oxidative stress, and inflammation [12]. However, the low solubility, poor bioavailability, and short half-life limit further application of puerarin [10]. Thus, improving its oral bioavailability has attracted widespread attention. In recent years, a series of studies on approaches to increasing the solubility and bioavailability of puerarin have been reported, including microemulsion drug delivery systems, nanotechnologies, and modifications of puerarin [13,14].

Therefore, the aims of this review are to provide an overview of the effect of puerarin on the regulation of glucose and lipid metabolism, including its mechanisms of action, and to summarize the potential solutions to the limitations of puerarin for further application. We manually searched original and review articles in English databases for possibly related studies. The eligible references were based on the following criteria: (1) studies that used puerarin as keyword; (2) studies including the effect of puerarin on MetS in animal models or tissue cells; (3) studies that reported bioavailability issues of puerarin.

## 2. Effect of Puerarin on Glucose and Lipid Metabolism

**Figure 1:**
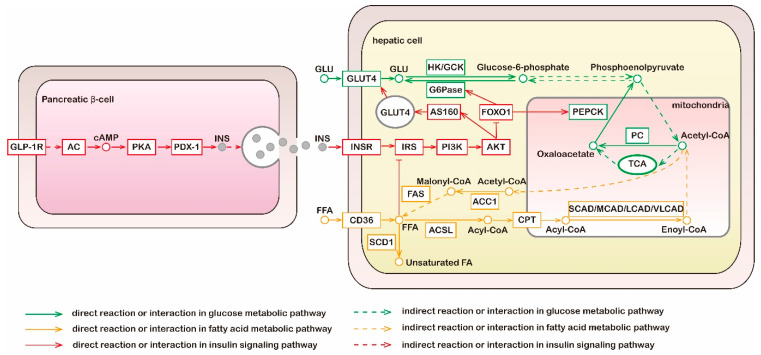
Metabolic and signaling pathways of glucose, fatty acid, and insulin. Liver is the main organ of gluconeogenesis; liver and adipose tissue are main sites of fatty acid synthesis; the above two metabolic pathways do not occur in muscle tissue. Green lines, molecular interaction or relation in glucose metabolic pathway; yellow lines, molecular interaction or relation in fatty acid metabolic pathway; red lines, molecular interaction or relation in insulin signaling pathway; dotted lines, indirect reaction or interaction. GLP-1R, glucagon-like peptide 1 receptor; AC, adenylate cyclase; PKA, protein kinase A; PDX-1, pancreatic and duodenal homeobox 1; INS, insulin; GLU, glucose; GLUT4, glucose transporters 4; HK, hexokinase; GCK, glucokinase; G6Pase, glucose-6-phosphatase; TCA, tricarboxylic acid cycle; PC, pyruvate carboxylase; PEPCK, phosphoenolpyruvate carboxykinase; INSR, insulin receptor; IRS, insulin receptor substrates; PI3K, phosphatidylinositol 3-kinase; AKT, protein kinase B; SCD1, stearoyl-CoA isomerase 1; FFA, free fatty acid; ACSL, long-chain acyl-CoA synthetase; CPT, carnitine palmitoyl transferase; CAD, acyl-CoA dehydrogenases; ACC1, acetyl-CoA carboxylase 1; FAS, fatty acid synthase.

According to current knowledge, glucose metabolism is tightly linked with lipid metabolism (Figure 1). Sugars and lipids are metabolized via a series of reactions in various tissues and organs to produce a common metabolite, acetyl-CoA, which can be fully oxidized in the tricarboxylic acid (TCA) cycle and generates an abundance of energy [15,16]. Under conditions of carbohydrate excess, insulin resistance may be induced, and acetyl-CoA is directed from mitochondria to the cytosol for FA synthesis [16,17]. Rising circulating plasma FA levels can impair insulin’s ability to suppress hepatic gluconeogenesis [18]. Evidence has shown that puerarin has effects on the regulation of sugar and lipid metabolism and these effects are associated with multiple metabolic and signaling pathways [11,19]. Further details of the specific efficacy and mode of action are discussed below.

### 2.1. Mechanism of Glucose Metabolism Regulation

The results of many studies have proven the beneficial effects of puerarin on glucose metabolism. Puerarin treatment for four weeks observably decreased serum glucose and glycated hemoglobin (HbA1C) in T2DM rats [20]. Oral glucose tolerance and intraperitoneal insulin tolerance tests revealed that glucose and insulin intolerances in rats fed with a high-fat diet (HFD) were effectively suppressed by puerarin administration [21]. Moreover, the glucose uptake of the puerarin (30 μg/mL)-treated cells was 17% higher than that of high-glucose-induced IR cells [22].

In recent years, an amount of literature has been published on the anti-hyperglycemia effects of puerarin (Table 1). Here, we discuss the underlying mechanisms and molecular targets in the hypoglycemic effect of puerarin, focusing on carbohydrate hydrolyzing enzymes, glucose transporters, hepatic gluconeogenesis, insulin secretion, and insulin resistance.

#### 2.1.1. Effect of Inhibiting α-Amylase

Starch is a main dietary source of glucose and must be digested by a combination of enzymes, including α-amylases, to produce monosaccharides which can be absorbed through the intestinal wall [42]. Therefore, inhibiting α-amylase can slow down degradation of starch and control the level of serum glucose [43]. An inhibitory effect of puerarin on α-amylases has been observed in some studies, and the inhibitory rate was 30.76% at 48 μM on 10 mg/mL potato starch [44,45].

#### 2.1.2. Effect of Increasing GLUT4-Mediated Glucose Uptake

A family of glucose transporters (GLUTs) mediates glucose transport across the cell membrane, and GLUT4 is the most abundant GLUT isoform in muscle and adipose tissue responsible for insulin-stimulated glucose uptake [46]. Hsu and colleagues found that the mRNA and protein levels of GLUT4 in soleus muscle of STZ-induced diabetic rats were, respectively, 49% and 57% lower than those of normal rats and intravenous injection of puerarin (15.0 mg/kg) three times daily for three days led to a marked increase in both mRNA and protein levels of GLUT4 [23]. A similar action was also found in differentiating 3T3-L1 cells, for which treatment with 100 μM puerarin significantly upregulated the expression of GLUT4 mRNA [24]. Additionally, glucose uptake is not only related to GLUT4 gene expression, but also related to GLUT4 transposition. Insulin excites the movement of GLUT4-containing vesicles from cytoplasm toward cell membrane [47]. Hence, the GLUT4 transposition obstacle is regarded as one of the main causes of IR. In the study of Zhao and Zhou, GLUT4 transposition under insulin stimulation and puerarin intervention was observed by an immunofluorescence method. Their result confirmed that the transposition obstacle did happen in the free fatty acid (FFA)-induced IR model of 3T3-L1 lipocytes and that puerarin showed a promoting effect on GLUT4 transposition [25].

It is thought that the regulation of GLUT4 translocation is associated with the activation of the phosphatidylinositol 3-kinase (PI3K)–protein kinase B (Akt) pathway. Akt can phosphorylate its substrate AS160 and subsequently promotes GLUT4 translocation [48]. Reduced phosphorylation of Akt and AS160 would ultimately result in a reduced glucose uptake. Chen and colleagues assessed whether puerarin regulated the phosphorylation of Akt and AS160, as well as the protein level of total and membrane GLUT4 in soleus muscles of HFD/STZ-induced diabetic rats and 0.75 mM palmitate-incubated L6 cells. The results indicated that the contents of total and membrane GLUT4, as well as the rates of p-AS160/AS160 and p-Akt/Akt, in the puerarin treatment group were obviously higher than those in the model group, both in vivo and in vitro [26]. Taken together, this evidence suggested that puerarin could promote GLUT4-mediated glucose uptake by elevating GLUT4 mRNA and protein expression and upregulating the phosphorylation of Akt and AS160.

#### 2.1.3. Effect of Suppressing Gluconeogenesis

Endogenous glucose, which provides energy for tissues during periods of starvation, can be generated through the gluconeogenesis pathway. Gluconeogenesis occurs mainly in the liver and is regulated by various hormones including insulin [49]. Insulin resistance, a characteristic of T2DM, enhances endogenous glucose production and glucose release to the blood, and thus causes blood glucose elevation [50]. Phosphoenolpyruvate carboxykinase (PEPCK) and glucose-6-phosphatase (G6Pase), as rate-limiting enzymes, are the keys to regulating gluconeogenesis [51]. In Hou’s study, to clarify the effect of puerarin on gluconeogenesis, a rat model of T2DM was induced by feeding a high-fat high-sucrose (HFHS) diet along with intraperitoneal injection of STZ. The results showed that oral administration of puerarin at 100 mg/kg/day reduced the mRNA expression of PEPCK and G6Pase in liver compared with T2DM rats [27].

FOXO1 is a member of the forkhead transcription factors, which plays an important role in driving the expression of PEPCK and G6Pase. Early findings revealed that there were three phosphorylation sites in FOXO1, which could be phosphorylated by Akt. Phosphorylation of these sites results in the exclusion of FOXO1 from the nucleus, and further suppresses the key enzymes of gluconeogenesis [52]. In previous research, a reduction in pFOXO1/FOXO1 and a rise in PEPCK and G6Pase protein expression were observed in the liver of HFD/STZ-induced T2DM rats and palmitic acid-induced IR HepG2 cells. However, puerarin exerted an elevating effect on pFOXO1/FOXO1 and suppressed protein expression of PEPCK and G6Pase in the rats’ liver tissues throughout the four weeks of the experiment. The results in vitro are consistent with those in vivo [20].

In addition, puerarin also significantly upregulated the protein expression of PI3K and the ratio of pAkt/Akt in T2DM rats and isolated cells [36,38]. Moreover, the administration of an inhibitor of PI3K and Akt could reverse the regulatory effect of puerarin on gene expression, which proved that puerarin’s inhibition of FOXO1 phosphorylation occurred via activation of the PI3K–Akt signaling pathway [20]. All these results clearly showed that gluconeogenesis could be suppressed by puerarin through reduction of PEPCK and G6Pase expression, and that FOXO1 is an important target for mediating effects of the PI3K–Akt pathway on gene expression downstream.

#### 2.1.4. Effect of Promoting Insulin Secretion

Insulin is a key hormone of blood glucose regulation and is secreted by pancreatic β-cells [53]. Evidence has proved that puerarin contributes to a decrease in β-cell apoptosis and an increase in β-cell proliferation. TUNEL staining was conducted to investigate β-cell apoptosis in pancreatic sections. As shown in Yang’s study, the number of positive β-cells increased 7.6-fold in HFD mice and 26.4-fold in db/db mice compared to controls. Treatment with puerarin (oral gavage at dosage of 150 mg/kg daily for 35 days) reduced positive β-cells by 52.4% and 71.4%, respectively, in HFD and db/db mice [28]. The prevention effect of puerarin on β-cell apoptosis may be associated with caspase-3, which plays an important role in the apoptosis pathway. It has been observed that puerarin effectively abolished the elevated protein expression of caspase-3 in the pancreas of HFD/STZ-induced T2DM mice [29]. Ki-67 staining was applied to detect the impact of puerarin on β-cell proliferation. Wang and colleagues found that HFD treatment for 12 weeks impaired β-cell proliferation in mice, and positive β-cells increased markedly in mice treated with puerarin [30].

In other aspects, puerarin has the potential to stimulate insulin synthesis. A glucose-stimulated insulin secretion (GSIS) assay revealed that high glucose incubation strongly impaired insulin secretion from islets in mice, whereas puerarin induced a notable increase in insulin release. Meanwhile, mRNA levels of glucagon-like peptide 1 receptor (GLP-1R) and insulin, as well as protein levels of GLP-1R and pancreatic and duodenal homeobox 1 (PDX-1), were significantly decreased by high glucose in islets [28]. GLP-1 influences insulin secretion by activation of adenylate cyclase (AC), which results in elevation of cAMP and then leads to activation of protein kinase A (PKA) [54]. PDX-1 is a transcription factor in β-cells that can regulate insulin gene transcription, and can be activated by PKA [55]. The results also showed that the mRNA levels of GLP-1R, PDX-1, and insulin, as well as protein levels of GLP-1R and PDX-1, were significantly increased by puerarin. Furthermore, the promoting effect of puerarin on insulin secretion was enhanced by an agonist of GLP-1R and inhibited by an antagonist of GLP-1R, which confirmed the vital role of GLP-1R in the action of puerarin [28]. Overall, GLP-1R signaling is possibly the foremost signaling pathway in insulin biosynthesis upregulated by puerarin; however, more detailed mechanisms need to be studied further.

#### 2.1.5. Effect of Improving Insulin Resistance

The insulin signal is transduced through binding between insulin and its cell surface receptor, which results in phosphorylation of the insulin receptor (InsR) and activation of receptor tyrosine kinases. InsR tyrosine kinases catalyze tyrosine phosphorylation of insulin receptor substrate 1 (IRS-1), which can bind to a subunit of PI3K and activate PI3K [56]. FAs were thought to affect the insulin signaling pathway in muscles and myocytes. FA metabolites induce serine phosphorylation of IRS-1 via activation of serine/threonine kinases, which causes IRS-1 to lose the ability to associate with and activate PI3K [57]. Studies using a euglycemic–hyperinsulinemic clamp have demonstrated that lipid infusion markedly reduced IRS-1 tyrosine phosphorylation, PI3K activity, and Akt phosphorylation in skeletal muscle, and palmitic acid treatment decreased expression and activity of InsR, as well as phosphorylation of IRS-1 (at tyrosine residues) and Akt, in soleus muscle and myocytes [58].

The condition where cells become resistant to the effects of insulin is called insulin resistance and leads to relative insulin deficiency, which is a critical factor in the progression of T2DM [59]. A 2-week treatment of puerarin significantly increased InsR mRNA expression in skeletal muscle of STZ-induced diabetic mice in a dose-dependent manner [31]. Puerarin-treated HFD/STZ-induced diabetic rats showed significant upregulation of IRS-1 protein content and an increase in the ratios of pInsR(Tyr^1150/1151^)/InsR and pIRS-1(Tyr^612^)/IRS-1. Similarly, the levels of mRNA and of IRS-1 protein in palmitate-induced IR L6 cells treated by puerarin were obviously higher than that of model cells [26]. These results suggested that the regulation effect of puerarin on the PI3K–Akt signaling pathway and its downstream proteins may be achieved by improving insulin sensitivity.

### 2.2. Mechanism of Regulating Lipid Metabolism

The beneficial effects of puerarin on lipid metabolism have been proved by the results of many studies (Table 1). Diabetic rats treated with puerarin had lower serum triglyceride (TG) and total cholesterol (TC) levels than diabetic model rats [32]. Similarly, a significant decrease, compared with HFD-fed mice, was found in serum and hepatic TG and TC levels of mice fed an HFD supplemented with puerarin [33]. In addition, puerarin could also reduce the serum level of FFA in diabetic rats and the FFA content of IR muscle cells [34].

This section of the present review highlights the mode of action of the effects of puerarin on lipid metabolism, including the following mechanisms: suppressing lipoprotein lipolysis, decreasing FA uptake, promoting FA degradation, and inhibiting FA and cholesterol synthesis.

#### 2.2.1. Effect of Suppressing Lipoprotein Lipolysis

Lipoprotein lipase (LPL) hydrolyzes TGs, in the cores of chylomicrons and VLDLs, releasing FFA [60]. As a target gene of PPARs, LPL can be upregulated by PPARγ [61]. Puerarin (20 μM) treatment for 6 days significantly decreased mRNA levels of LPL by 52.30% and PPARγ by 57.5%, compared with levels in the cells induced to undergo adipogenic differentiation without puerarin treatment [35]. These detected changes suggested that puerarin suppressed FA release by downregulating the expression of a lipolysis-related gene.

#### 2.2.2. Effect of Decreasing FA Uptake

CD36 and fatty acid binding protein (FABP) mediate FA uptake into myocytes and adipocytes, and their expressions are regulated by PPARs [62]. Although HFD/STZ treatment did not cause marked differences in total CD36 level, membrane levels of CD36 in the muscle of diabetic rats were significantly increased in comparison with that of controls [34]. In vitro study revealed a similar finding: that myotubes treated with palmitate had obviously higher membrane levels of CD36 than control cells. Treatment with puerarin reversed these changes and led to a significant reduction in membrane CD36 levels both in diabetic rats and IR cells. Reduced intramyocellular lipids in puerarin-treated rats, shown by transmission electron microscopy images, confirmed the prevention effect of puerarin on lipid accumulation [34]. In addition, in the 3T3-L1 cells induced to undergo adipogenic differentiation, puerarin significantly inhibited lipid accumulation and decreased FABP4 mRNA and protein levels. Moreover, the mRNA and protein levels of the key transcription factor, PPARγ, were also suppressed by puerarin [35]. Taken together, puerarin could prevent FA uptake by depressing membrane levels of CD36 and gene expression of FABP4 and their transcription factor PPARγ.

#### 2.2.3. Effect of Promoting FA β-Oxidation

FA degradation is mainly accomplished via mitochondrial and the peroxisomal β-oxidation pathways. The oxidation of short- and medium-chain FAs mainly occurs in mitochondria, and very-long-chain FAs are first shortened in the peroxisomes before being further oxidized in the mitochondria [63]. FAs are activated through esterification with coenzyme A, which is catalyzed by long-chain acyl-CoA synthetase (ACSL); the fatty acyl-CoAs are then transported into peroxisomes and oxidized via four successive reactions, the first step of which is catalyzed by acyl-CoA oxidases (ACOXs). After several rounds of β-oxidation, the shortened acyl-CoAs enter mitochondria through a system of proteins including carnitine palmitoyl transferase 1 (CPT-1). β-oxidation in mitochondria is similar to that in peroxisomes but involves different enzymes for the first step, which is catalyzed by acyl-CoA dehydrogenases (SCAD, MCAD, LCAD, and VLCAD) [63]. All the genes of these proteins participating in FA degradation are the target genes of PPARs [61]. Puerarin restored the abnormalities of mitochondrial structure and the number of mitochondria in rat muscle tissue [34]. On the other hand, a study conducted by Wang et al. [36] revealed that puerarin significantly increased mRNA expression of CPT-1, MCAD, and ACOX, as well as mRNA and protein levels of PPARα in the liver of HFHS diet-fed mice. In another study, puerarin could reverse the decrease in LCAD, ACOX, and PPARδ mRNA levels and CPT-1 protein level in muscle of HFD/STZ-induced diabetic rats. In addition, ACSL and LCAD mRNA levels in muscle cells could also be elevated by puerarin [34]. This evidence confirmed that puerarin could protect mitochondria, where β-oxidation is carried out, and influence the expression of enzymes related to FA degradation by enhanced PPAR expression, which promoted the oxidation of FA.

#### 2.2.4. Effect of Inhibiting FA Synthesis

De novo synthesis of FAs is an important metabolic pathway in liver and adipose tissue; it is induced by high carbohydrate levels and is controlled by hormones. Acetyl-CoA carboxylase 1 (ACC1) catalyzes the rate-limiting step in FA synthesis, and it can be phosphorylated and inactivated by AMP-activated protein kinase (AMPK). Fatty acid synthase (FAS) is another key enzyme that has a close relationship with the rate of FA synthesis and its activity is regulated by transcription factor SREBP1-c, which can be inhibited by AMPK [64,65]. Stearoyl-CoA isomerase (SCD) is also a lipogenic enzyme regulated by SREBP1c; it catalyzes the introduction of a double bond into saturated FAs [63]. Previous research has shown that an HFHS diet greatly enhanced hepatic mRNA expression of ACC and FAS in mice and oral administration of 0.4 g/kg/day puerarin significantly downregulated ACC and FAS mRNA levels [36]. In C2C12 cells, the phosphorylation of ACC and AMPK was markedly increased by 20 μM puerarin, which indicated an activation of AMPK and inhibition of ACC [37]. Xu et al. [38] found that puerarin reversed the decrease in ACC and AMPK phosphorylation in HepG2 cells treated with induction media containing FFA and fructose; treatment with an AMPK inhibitor largely abolished the effect of puerarin, which further indicated that puerarin exerted its ACC inhibition effect by action on the AMPK pathway. In addition, puerarin reduced the mRNA and protein levels of FAS and SREBP1-c in both oleic acid-treated HepG2 cells and liver tissues of HFHS diet-fed mice [36,39]. SCD1 and SREBP1c mRNA expression in the liver of HFHS/STZ-induced diabetic rats was also significantly downregulated by puerarin [27]. In short, puerarin inhibits de novo synthesis of FA by inactivating ACC and downregulating FAS and SCD expression, and the AMPK pathway is required for this modulation effect.

#### 2.2.5. Effect of Inhibiting Cholesterol Synthesis

HMGCR, as the rate-limiting enzyme of cholesterol synthesis, can be phosphorylated and inactivated by AMPK [64,66]. The in vitro experiment of Xu et al. [38] indicated that high-fat high-fructose challenge markedly elevated HMGCR mRNA expression, whereas puerarin could significantly suppress the mRNA level of HMGCR and increase the phosphorylation of AMPK.

### 2.3. Oxidative Stress and Inflammation

Hyperglycemia has been considered one of the primary contributors to chronic and sustained oxidative stress. Hyperglycemic conditions can lead to increased generation of reactive oxygen species (ROS) and reduced activity of antioxidant enzymes [67]. Lipid accumulation can also promote the production of ROS and induce oxidative stress [68]. It is thought that oxidative stress can mediate mitochondrial dysfunction and stimulate inflammatory responses. Additionally, both a high level of ROS and chronic systemic inflammation contribute to insulin resistance [67]. Puerarin administration significantly lowered peroxidation end product malondialdehyde (MDA) content and ROS level and improved the antioxidant capacity by elevating glutathione (GSH) level and superoxide dismutase (SOD) activity in diabetic mice [40,41]. The regulatory effect of puerarin on inflammation was confirmed by the changes in inflammatory factors. The results showed that IL-1β, IL-6, and TNF-α in diabetic rat liver tissue are reduced by puerarin [27].

## 3. Approaches to Improving Oral Bioavailability of Puerarin

Puerarin has a low water solubility of 0.46 mg/mL. In pharmacokinetic studies, using oral dosing in rat models, 5 mg/kg puerarin has a half-life (t_1/2_) of 0.88 h and a maximum plasma concentration (C_max_) of 145.47 μg/L. The absolute oral bioavailability of puerarin was 7.5% [69]. The poor bioavailability hinders the clinical performance of puerarin; thus, developing appropriate formulations of puerarin to enhance oral bioavailability has important significance.

### 3.1. Microemulsion and Self-Microemulsifying Drug Delivery Systems

A microemulsion (ME) is a thermodynamically stable dispersion system consisting of a water phase, an oil phase, a surfactant, and a cosurfactant [70]. MEs can enhance the bioavailability of poorly water-soluble drugs. Wu et al. [71] developed a new ME of puerarin based on the phospholipid complex technique. As a result, puerarin–phospholipid complex (PPC) ME exhibited the highest C_max_, among puerarin, PPC, puerarin–ME (Pue-ME), and PPC-ME, in rats. In addition, compared to puerarin, the relative bioavailabilities of Pue-ME and PPC-ME were 2.52- and 3.16-fold higher, respectively. In the study of Liao, N-trimethyl chitosan (TMC) was added to the formulation to increase the oral bioavailability of puerarin. Pharmacokinetic studies demonstrated that, after oral administration of puerarin–TMC-modified microemulsion (Pue-TME) and Pue-ME to rats, C_max_ and t_1/2_ were both increased, compared to puerarin suspension as control. The relative bioavailabilities of Pue-TME and Pue-ME were, respectively, 6.8- and 1.2-fold higher than control, which indicated a significant bioavailability promotion effect of TMC-MEs [72].

A self-microemulsifying drug delivery system (SMEDDS) is a mixture of oils, surfactants, and cosurfactants, and has been proven to be one of the most effective approaches to improving the solubility and oral absorption of drugs. Compared with MEs, one of the greatest advantages of a SMEDDS is that it can form ME in aqueous media spontaneously [73]. Cheng et al. established a method for preparing a puerarin solid SMEDDS using a spherical crystallization technique. The results of pharmacokinetic experiments in rats suggested that, compared to the suspension, the relative bioavailability levels of the liquid SMEDDS and solid SMEDDS were increased 23.23-fold and 27.03-fold, respectively; there was no significant difference between two self-microemulsion groups. However, the solid SMEDDS had significant effects on decreasing the rate of elimination of puerarin in comparison with the liquid SMEDDS. The t_1/2_ of the solid SMEDDS was 4.7 times that of the suspension, and 1.91 times that of the liquid SMEDDS [74]. In the study of Yi, a SMEDDS coloading borneol and puerarin was prepared to investigate its effects on improving the oral absorption and brain penetration of puerarin in mice. The results suggested that the SMEDDS significantly enhanced the oral absorption of puerarin, and the bioavailability of the SMEDDS relative to nanocrystal suspension and inclusion compound solution was 173.5% and 227.8%, respectively [75].

### 3.2. Nanoparticles and Nanocrystals

Nanoparticles (NPs) as drug carriers are a combination of nanotechnology and modern medicine. NPs are the focus of attention in studies on drug delivery technologies because of the advantages of improving bioavailability [76]. Yan et al. prepared puerarin nanoparticles (Pue-CS/TPP-NPs) through the ionic crosslinking of chitosan (CS) and sodium tripolyphosphate (TPP). Rat pharmacokinetic results showed that Pue-CS/TPP-NPs increased bioavailability by 443.3% relative to puerarin and the maximum release time of the nanoparticles shifted back, indicating that the nanoparticles had a sustained-release effect [77]. Chen et al. developed spherical NPs from six-armed star-shaped poly(lactide-co-glycolide) (6-s-PLGA) NPs that could be used to encapsulate puerarin, and then conducted a pharmacokinetic study in rats. The results revealed that, relative to unformulated puerarin, the t_1/2_ of puerarin NPs rose 5.00-fold, and the bioavailability of puerarin NPs was 10.52-fold higher. This significant improvement in bioavailability may be due to the gradual release of puerarin from NPs, which protects the drug from being eliminated [78].

Nanocrystals (NCs) are also one of the technologies that can overcome undesirable oral bioavailability of drugs with poor water solubility. A nanocrystal system uses different stabilizers to stabilize the nanoscaled drug crystals, thus improving the efficacy of the drug delivery system [79]. In Xiong’s study, puerarin nanocrystals (Pue-NCs) were prepared by an antisolvent precipitation method, and pharmacokinetic analysis was carried out to determine the plasma concentrations of puerarin. When compared with the puerarin suspension, the C_max_ and relative bioavailability in the plasma of rats following oral administration of Pue-NCs were enhanced 7.14-fold and 4.80-fold, respectively [80]. Tu et al. constructed Pue-NCs with ultra-small particle sizes (below 50 nm) by high-pressure homogenization. The reduced particle size contributed to a higher solubility, thus leading to enhanced bioavailability. The blood concentration–time curves of puerarin suspension and NCs showed that the absolute bioavailability of Pue-NCs was 35.28%, which was 11.54-fold higher than that of the puerarin suspension [81].

### 3.3. Glycosylation Modification

In recent years, studies have been carried out to improve the bioactivity of puerarin via structural modifications. Glycosylation is emerging as an efficient approach to increasing the water solubility, bioavailability, and other physicochemical and biological properties of flavonoids [82]. Huang and colleagues synthesized three puerarin glucosides by using a cyclodextrin glucanotransferase from *Bacillus licheniformis* with α-cyclodextrin as the sugar donor. Three puerarin glucosides showed 15.6, 100.9, and 179.1 times more water solubility, respectively, than puerarin [83]. A method for the isolation and purification of puerarin glycosides from the crude products after enzymatic glycosylation of puerarin by high-speed counter-current chromatography was established by Wu and colleagues. β-d-fructofuranosyl-(2→6)-puerarin was one of four fructosyl puerarins that were successfully purified, and it exhibited the improved pharmacokinetic behavior of having a longer elimination half-time than puerarin in the blood of rats [84].

## 4. Conclusions and Future Perspectives

The consumption of foods containing excessive sugar and fat is increasing year by year. This unhealthy dietary pattern leads to a series of metabolic disorders. The existing pharmacotherapy for MetS is considered to have serious side effects. Hence, natural bioactive ingredients from plants are attractive alternatives to classical treatment.

**Figure 2:**
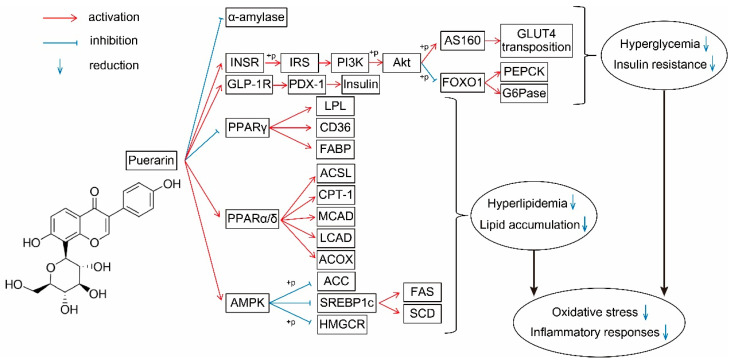
Effect of puerarin on sugar and lipid metabolism and its molecular mechanisms. Red lines, activation; blue lines, inhibition; down arrows, reduction. INSR, insulin receptor; IRS, insulin receptor substrates; PI3K, phosphatidylinositol 3-kinase; AKT, protein kinase B; GLUT4, glucose transporter 4; G6Pase, glucose-6-phosphatase; PEPCK, phosphoenolpyruvate carboxykinase; GLP-1R, glucagon-like peptide 1 receptor; PDX-1, pancreatic and duodenal homeobox 1; PPAR, peroxisome proliferator-activated receptors; LPL, lipoprotein lipase; FABP, fatty acid binding protein; ACSL, long-chain acyl-CoA synthetase; CPT, carnitine palmitoyl transferase; CAD, acyl-CoA dehydrogenases; ACOX, acyl-CoA oxidases; ACC, acetyl-CoA carboxylase; HMGCR, 3-hydroxy-3-methylglutaryl-CoA reductase; FAS, fatty acid synthase; SCD1, stearoyl-CoA isomerase 1.

**Figure 3:**
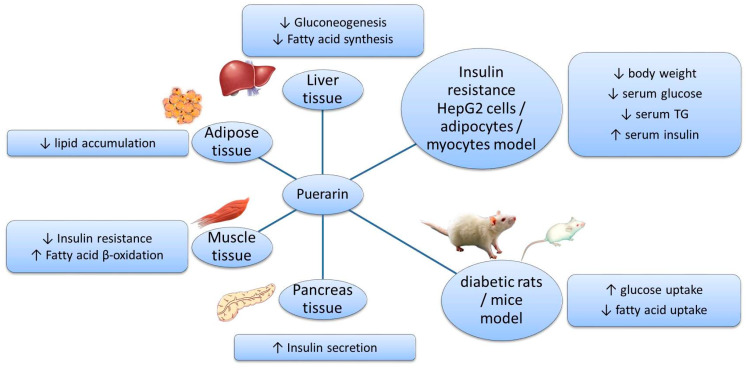
Effects of puerarin in different models and tissues.

Puerarin is a natural ingredient isolated from *Pueraria lobata*. There is evidence supporting the beneficial effects of puerarin on the regulation of sugar and lipid metabolism (Figure 2): suppressing the release of glucose and FFA by inhibiting related enzymes; regulating the transport of glucose and FA; decreasing the synthesis of glucose and FA by acting on PI3K–Akt and AMPK signaling pathways; promoting β-oxidation by protecting mitochondria and acting on the PPAR signaling pathway; improving insulin secretion and sensitivity; and alleviating oxidative stress and inflammatory responses. These effects have been demonstrated in various models and tissues (Figure 3). However, the application of puerarin is limited by its poor solubility and absorption. To improve the bioavailability of puerarin, drug delivery systems have been developed and various preparation technologies, such as microemulsions, SMEDDSs, nanoparticles, nanocrystals, and glycosylation, have been applied.

Nevertheless, there are still some deficiencies in the anti-MetS literature on puerarin. The current understanding of the role of puerarin in the treatment of sugar and lipid metabolic disorders is based on research at the animal or cell level, but clinical data are very limited. Furthermore, the potential of puerarin to regulate the combination of sugar metabolism and lipid metabolism needs more investigation. In addition, it remains to be further studied whether the methods to improve bioavailability mentioned above affect the biological activities of puerarin.

In summary, puerarin has excellent effects on the regulation of metabolic disorders of sugar and lipid metabolism and its clinical applications appear promising.

## Figures and Tables

**Table 1:** Effect of puerarin on glucose and lipid metabolism.

Model	Administration	Results	Reference
HFD/STZ-induced diabetic rats	oral administration of puerarin at 300 mg/kg/day for 4 weeks	↓ body weight ↓ blood glucose ↑ insulin ↓ HbA1C ↓ IPGTT ↓ IPPTT ↓ TG ↑ pFOXO1/FOXO1 ↓ mRNA and protein expression of PEPCK, G6Pase in liver ↑ protein expression of PI3K in liver ↑ pAkt/Akt	[20]
PA-treated HepG2 cells	incubation with 100 μmol/L puerarin	↑ protein expression of PI3K ↑ pAkt/Akt ↑ pFOXO1/FOXO1 ↓ protein expression of PEPCK, G6Pase	[20]
HFD-fed rats	intraperitoneal administration of puerarin at 100 and 200 mg/kg/day for 8 weeks	↓ body weight ↓ OGTT ↓ ITT ↓ serum leptin and resistin ↓ mRNA expression of leptin, resistin in adipose tissue	[21]
high-glucose-induced IR adipocytes	incubation with puerarin (10, 30 μg/mL)	↑ glucose uptake	[22]
STZ-induced diabetic rats	intravenous injection of puerarin at 15 mg/kg three times daily for 3 days	↓ plasma glucose ↓ IVGCT ↑ glucose uptake in muscle ↑ mRNA and protein expression of GLUT4	[23]
3T3-L1 adipocytes	incubation with puerarin (10, 100 μM)	↑ mRNA expression of PPARγ and FABP ↑ mRNA expression of GLUT4 ↑ mRNA expression of adiponectin ↑ mRNA expression and enzyme activity of G6PDH ↑ mRNAs expression of GR and CAT ↓ ROS	[24]
FFA-induced IR 3T3-L1 lipocyte	incubation with puerarin (1.5, 0.75 mg/mL) for 48 h	↑ glucose transportation ↑ GLUT4 transposition ↓ mRNA expression of PPARγ	[25]
HFD/STZ-induced diabetic rats	intraperitoneal injection of puerarin (100 mg/kg) for 4 weeks	↓ IPGTT ↓ FBG, GSP ↑ plasma insulin ↑ protein expression of IRS-1 in muscle ↑ pIR/IR, pIRS-1/IRS-1, pAkt/Akt ↑ p AS160/AS160 ↑ total and membrane GLUT4	[26]
palmitate-induced IR L6 myotubes	incubation with 0.3 mM puerarin for 24 h	↑ glucose uptake ↑ pAkt/Akt ↑ pAS160/AS160 ↑ membrane GLUT4 ↑ protein expression of IRS-1	[26]
HFHS/STZ-induced diabetic rats	oral administration of puerarin at 100 mg/kg/day	↓ FBG, fructosamine ↓ ALT, AST ↓ serum TG, TC, LDL ↓ mRNA expression of PEPCK, G6Pase in liver ↑ hepatic lipase activity in liver ↓ mRNA expression of SREBP1c, SCD1 in liver ↓ serum MDA, 8-OHdG ↑ T-AOC, CAT in liver ↑ serum SOD ↓ mRNA expression of IL-1β, IL-6, TNF-α, MCP-1 in liver	[27]
HFD-fed mice	oral administration of puerarin at 150 mg/kg daily for 35 days	↓ body weight ↓ FBG ↓ IPGTT ↑ serum insulin ↑ β-cell mass ↓ serum TC ↓ serum leptin	[28]
*db*/*db* mice	oral administration of puerarin at 150 mg/kg daily for 55 days	↓ body weight ↓ FBG ↓ IPGTT ↑ serum insulin ↑ β-cell mass ↓ serum TG, TC ↑ serum adiponectin	[28]
high-glucose-cultured mouse islets	treatment with puerarin (50 μM)	↑ mRNA expression of GLP-1R, TCF7L2, PDX-1, insulin ↑ GSIS ↑ protein expression of GLP-1R, PDX-1 ↓ protein expression of caspase-3 ↑ p-FOXO1, p-Akt	[28]
high-glucose-treated Min6 cells	treatment with puerarin	↑ membrane/cytosolic ratio of GLP-1R	[28]
HFD/STZ-induced diabetic mice	oral administration of puerarin at 80 mg/kg for 15 days	↓ FBG ↓ serum insulin ↓ serum TC, TG, LDL ↑ serum HDL ↓ TUNEL-positive cells in pancreas ↓ protein expression of caspase-3, 8, 9 and AIF in pancreas	[29]
HFD-fed mice	oral administration of puerarin at 150 and 300 mg/kg/day for 20 days	↓ FBG ↓ body weight ↓ OGTT ↑ serum insulin ↓ serum glucagon ↓ HOMA-IR ↑ HOMA-β ↑ Ki-67 positive β-cells ↑ positive PDX-1 and Ngn3 expressions ↑ protein expression of GLP-1R in isolated cultured mouse pancreatic ductal cells	[30]
STZ-induced diabetic mice	oral administration of puerarin at 20, 40, 80 mg/kg for 14 days	↑ body weight ↓ FBG ↑ serum insulin ↓ serum TC, TG, LDL ↑ serum HDL ↑ protein expression of IRS-1, GLP-1R in pancreas ↑ mRNAs expression of InsR, PPARa	[31]
STZ-induced diabetic rats	treatment with puerarin (140, 200 mg/kg/day)	↑ body weight ↓ FBG ↑ serum insulin ↓ serum TC, TG ↑ SOD, CAT, GSH-Px in kidneys ↓ MDA, NO in kidneys ↓ plasma IFN-γ ↑ plasma IL-4	[32]
HFD-fed mice	0.2%, 0.4%, or 0.8% puerarin-supplemented HFD	↓ body weight ↓ serum TG, TC, and leptin ↓ hepatic TG ↓ FAS activity in liver ↑ CAT, HSL, and AMPK activity in liver ↓ mRNA expression of PPARγ in liver ↑ mRNA expression of CAT, HSL, ACO in liver ↓ protein expressions of PPARγ in liver ↑ protein expressions of p-AMPK, HSL, and p-HSL in liver	[33]
HFD/STZ-induced diabetic rats	intraperitoneal injection of puerarin (100 mg/kg) for 4 weeks	↓ body weight ↓ serum TC, TG ↑ serum SOD ↓ serum MDA ↓ membrane CD36 in skeletal muscle ↑ protein expressions of CPT-1 in skeletal muscle ↑ p-AMPK/AMPK, p-ACC/ACC ↑ mRNA expressions of LCAD, ACOX, PPARδ in skeletal muscle ↑ number of mitochondria	[34]
0.75 mM palmitate-cultured (24 h) L6 myotubes	incubation with puerarin (0.3 mM) for 24 h	↑ pAkt/Akt ↓ membrane CD36 ↑ mRNA expressions of ACSL, LACD ↑ p-ACC/ACC ↓ FFA	[34]
3T3-L1 cells with adipocyte differentiation induction	treatment with puerarin (10 and 20 μM)	↓ lipid accumulation ↓ mRNA expressions of PPARγ, C/ebpα, LPL, Fabp4 ↓ protein expressions of PPARγ, C/EBPα, FABP4 ↑ p-GSK-3β/GSK-3β, p-Akt/Akt	[35]
HFHS-induced NAFLD mice	oral administration of puerarin at 0.2, 0.4 g/kg for 18 weeks	↓ body weight ↓ ALT, AST in liver ↓ IFN-γ, IL-1β, IL-6 in liver ↑ p-PI3K, p-Akt in liver ↓ mRNA expressions of SREBP-1, ACC, FAS in liver ↑ mRNA and protein expression of PPARα in liver ↑ NAD+, ATP in liver ↑ mRNA expression of ACOX, CPT-1, MCAD	[36]
differentiated C2C12 myotubes	treatment with puerarin (10 or 20 µM) for 24 h	↑ p-AMPK/AMPK, p-ACC/ACC ↑ ATP	[37]
high-fat and -fructose diet-fed rats	0.2% puerarin-supplemented high-fat and -fructose diet for 16 weeks	↓ body weight ↓ serum TC, TG, LDL ↑ serum HDL ↓ hepatic TC, TG ↓ FBG, insulin ↓ OGTT, ITT ↑ serum T-AOC ↓ serum MDA ↑ p-AMPK/AMPK, p-Akt/Akt ↑ protein expression of PI3K in liver	[38]
HepG2 cells cultured with 10 mM glucose, 15 mM fructose, and 0.5 mM of FFA	induction with puerarin (75 μM and 150 μM) for 24 h	↓ TG ↑ p-AMPK/AMPK, p-ACC/ACC ↓ mRNA expression of SREBP1c, FAS, SCD, HMGCR ↑ glucose uptake ↑ mRNA expression of GLUT4 ↑ protein expression of PI3K ↑ p-Akt/Akt ↑ CAT	[38]
oleic acid-treated HepG2 cells	pre-treatment with puerarin (25, 50 and 100 µM) for 1 h	↓ TG, TC ↓ mRNA and protein expression of SREBP-1 and FAS ↑ mRNA and protein expression of PPARα ↑ p-AMPK/AMPK	[39]
STZ-induced diabetic mice	oral administration of puerarin at 100, 200, 400 mg/kg/day for 3 weeks	↑ body weight ↓ FBG, OGTT, fructosamine ↓ plasma MDA ↑ plasma SOD, GSH	[40]
HepG2 cells treated with insulin (3 × 10^−8^ mol/L)	treatment with 1 μmol/L puerarin	↑ glucose consumption ↑ p-AMPK/AMPK	[40]
HFD/STZ-induced diabetic mice	oral administration of puerarin at 200 mg/kg/day for 8 weeks	↑ body weight ↓ OGTT ↓ FBG, insulin ↓ serum TG, TC, LDL, FFA ↑ serum HDL ↑ SOD, GSH in liver ↓ MDA, ROS in liver ↑ protein expression of Nrf2, HO-1 ↑ protein expression of PI3K	[41]

Note: ↑, increase; ↓, decrease.

## Data Availability

Data is contained within the article.
